# Gastrointestinal discomfort and hypotension in a patient with *Reutealis trisperma* seeds intoxication: A case report

**DOI:** 10.1097/MD.0000000000028348

**Published:** 2021-12-23

**Authors:** Po-Min Chang, Yen-Yi Lee, Yen-Hung Wu

**Affiliations:** Department of Emergency Medicine, Kaohsiung Medical University Hospital, Kaohsiung Medical University, Kaohsiung, Taiwan.

**Keywords:** case report, gastrointestinal discomfort, hypotension, *Reutealis trisperma*

## Abstract

**Rationale:**

*Reutealis trisperma* is a plant belonging to the Euphorbiaceae family and *Reutealis* genus and is often mistaken for a plant of the genus *Aleurites*. Accidental ingestion of *R trisperma* seeds is relatively rare in Taiwan than that of *Vernicia fordii*. Mostly, the clinical course of *R trisperma* seed poisoning is similar to that of *V fordii* poisoning. Recent studies have shown that the median lethal dose 50 of *R trisperma* seeds in mice is approximately 4954 mg/kg. *R trisperma* seed extract has a significant effect on the autonomic nervous system by causing ptosis and disrupting breathing, and affects the central nervous system by reducing motor activity.

**Patient concerns:**

A 51-year-old man with underlying gout and hepatitis B picked several seeds of *R trisperma,* which he misidentified at chestnuts, at an elementary school. He prepared soup by boiling 3 to 4 seeds and consumed it. He experienced abdominal pain, vomiting, and watery diarrhea with hypotension.

**Diagnosis:**

*R trisperma* seeds intoxication.

**Interventions:**

The patient was given a soft diet, input and output were recorded, and intravenous fluid supplements were administered.

**Outcomes:**

The patient was discharged after 3 days of hospitalization, once a relatively stable condition was achieved.

**Lessons:**

Human poisoning by accidental consumption of *R trisperma* seeds is relatively rare in Taiwan. It may cause gastrointestinal symptoms and even hypotension. Patients can recover within 2 to 3 days of receiving proper treatment and intravenous fluid infusion.

## 1 Introduction

*Reutealis trisperma* is a plant belonging to the Euphorbiaceae family and *Reutealis* genus and is often mistaken for a plant of the genus *Aleurites*. It is also known as the Philippine tung, Banucalag nut, Soft lumbang, and Javillo. The Philippine tung tree is scattered throughout Taiwan; however, cases of accidental consumption of *R trisperma* are uncommon. Here, we present a rare case of accidental consumption of *R trisperma* in Taiwan. According to the regulations of the institutional review board of the Kaohsiung Medical University Hospital, ethical approval for this case report article is not required. Informed consent was obtained from the patient for the publication of this case report.

## 2 Case report

A 51-year-old man with underlying gout and hepatitis B picked several seeds of *R. trisperma,* which he misidentified at chestnuts, at an elementary school. He prepared soup by boiling 3 to 4 seeds and consumed it. However, 30 minutes after consumption, he experienced abdominal pain, vomiting, and watery diarrhea. His wife, who had eaten the same dinner with him but did not consume the soup, did not have the same symptoms. Therefore, he was immediately brought to the emergency department (ED).

During the triage at the ED, his initial vital signs showed a body temperature of 36.4°C, heart rate of 68 beats per minute, blood pressure of 78/46 mm Hg, Glasgow Coma Scale score 15, and clear consciousness. At the ED, the patient had persistent diarrhea, and vomiting with abdominal pain, including other symptoms, such as cold sweats and dizziness; however, there was no headache or limb weakness. There were no abnormal findings on physical examination. For suspected fluid depletion-induced hypotension, fluid supplements were administered immediately, and his blood pressure recovered soon. Blood examination showed leukocytosis (white blood cell: 12630/μL), predominantly neutrophilia (86.2%), and C-reactive protein level <0.2 mg/L. There was no obvious renal or liver dysfunction. Electrolytes, including sodium and potassium ions, were also within the normal range. Oliguria was present, and the urine was slightly cloudy and reddish; however, urine analysis did not reveal hematuria or pyuria.

The patient was admitted to the gastroenterology ward for further observation and treatment of the watery diarrhea and hypotension induced by *R trisperma* seed intoxication. The patient was given a soft diet, input and output were recorded, and intravenous fluid supplements were administered in the ward. After admission, no recurrent vomiting or watery diarrhea occurred. The patient was discharged after 3 days of hospitalization, once a relatively stable condition was achieved.

## 3 Discussion

The scientific name of *R trisperma* is *R trisperma* (Blanco) Airy Shaw or *Aleurites trisperma* (Blanco). *R trisperma* belongs to the Euphorbiaceae family and is a monotypic plant genus in the family Euphorbiaceae.

According to previous literature, plants belonging to the Euphorbiaceae family have varied biological behavior, such as antimycobacterial, cytotoxic, antimicrobial, anti-inflammatory, and antiviral effects, such as anti-HIV (in vitro).^[1–4]^*R trisperma* is endemic to the Philippines and is used as a timber species, although the International Union for Conservation of Nature has classified it with the conservation status “vulnerable.”^[5]^

*R trisperma* is a nonedible plant. Because of its oil content and high seed production capacity, it can be used to produce biodiesel. The seeds of this plant have a high oil content of up to 56%. In addition, it can grow well in unfavorable environments.^[6–8]^

Taiwan's tung tree is not a native but an exotic species. The most common ones include *Vernicia fordii*, *Vernicia montana*, and *R trisperma*. Every year in May, the tung tree's blossom season attracts many tourists to see the blooming snow-white tung tree flowers. In 1992 and 1994, 2 outbreaks of *V fordii* poisoning were reported in Taiwan.^[9]^ Dozens of students mistook the seeds of *V fordii* for chestnuts and consumed them. The 3 most common symptoms in patients with poisoning due to tung tree seeds are diarrhea, vomiting, and abdominal pain. Relatively serious cases occur more frequently among young individuals. However, all the symptoms subside within a day or 2 after treatment. Accidental ingestion of *R trisperma* seeds is relatively rare in Taiwan than that of *V fordii*. Mostly, the clinical course of *R trisperma* seed poisoning is similar to that of *V fordii* poisoning. At present, the mechanism of poisoning due to *R trisperma* seeds is still unclear. However, recent studies have shown that the median lethal dose 50 of *R trisperma* seeds in mice is approximately 4954 mg/kg. *R trisperma* seed extract has a significant effect on the autonomic nervous system by causing ptosis and disrupting breathing, and affects the central nervous system by reducing motor activity.^[10]^ As observed in our case, humans may have gastrointestinal symptoms, such as diarrhea, abdominal pain, and vomiting after ingesting *R trisperma* seeds. These gastrointestinal symptoms may even lead to hypotension. The patient recovered completely within 3 days of receiving symptomatic treatment and intravenous fluid supplementation.

## 4 Conclusion

Human poisoning by accidental consumption of *R trisperma* seeds is relatively rare in Taiwan. It may cause gastrointestinal symptoms and even hypotension. Patients can recover within 2 to 3 days of receiving proper treatment and intravenous fluid infusion.

## Author contributions

**Data curation:** Yen-Yi Lee.

**Visualization:** Yen-Yi Lee.

**Writing – original draft:** Po-Min Chang.

**Writing – review & editing:** Yen-Hung Wu.
